# Data on the influence of cold isostatic pre-compaction on mechanical properties of polycrystalline nickel sintered using Spark Plasma Sintering

**DOI:** 10.1016/j.dib.2017.01.009

**Published:** 2017-01-17

**Authors:** Guy-Daniel Dutel, Patrick Langlois, David Tingaud, Dominique Vrel, Guy Dirras

**Affiliations:** Université Paris 13, Sorbonne Paris Cité, LSPM, CNRS UPR 3407, 99 avenue Jean-Baptiste Clément, 93430 Villetaneuse, France

**Keywords:** Spark Plasma Sintering, Cold isostatic compaction, Nickel powders, Microstructure

## Abstract

Data regarding bulk polycrystalline nickel samples obtained by powder metallurgy using Spark Plasma Sintering (SPS) are presented, with a special emphasis on the influence of a cold isostatic pre-compaction on the resulting morphologies and subsequent mechanical properties. Three types of initial powders are used, nanometric powders, micrometric powders and a mixture of the formers. For each type of powder, the SPS cycle has been optimized for the powders without pre-compaction and the same cycle has been used to also sinter pre-compacted powders.

**Specifications Table**

**Table:** 

Subject area	*Physics*
More specific subject area	*Powder metallurgy*
Type of data	*Images, Figures*
How data was acquired	*SEM (Carl Zeiss Supra 40 VP-FEG), EBSD (TexSEM OIM 5 Software), compression tests (Deben*^*TM*^*Microtesting machine).*
Data format	*Analyzed*
Experimental factors	*Commercial powders of different grain sizes, nanometric and micrometric, or a mixture of them were sintered using Spark Plasma Sintering (SPS), either directly from loose powders or after a Cold Isostatic Pressing (CIP) step.*
Experimental features	*After polishing, samples were observed by SEM and EBSD, and compression tests were performed to determine mechanical characteristics of processed bulk materials.*
Data source location	*Laboratoire des Sciences des Procédés et des Matériaux, UPR 3407, 99 avenue Jean-Baptiste Clément, 93430 Villetaneuse, France.*
Data accessibility	*Data is with this article*

**Value of the data**

• The data describe the influence of a pre-compaction step on the final microstructure after SPS.
• The data presents the influence of the nature of the initial powders.
• The data provides information on the influence of these microstructures on the resulting mechanical properties.
• Pre-compaction by CIP followed by SPS procedure can serve as a mean for Σ3 grain boundaries engineering.
• Data shows that presence of Σ3 grain boundaries allows for a good compromise between compressive stress and strain to failure.

## 1 Data

Nanometric, micrometric and a 40% nanometric – 60% micrometric mixture of powders have been sintered using SPS, with an optional pre-compaction of the powders using Cold Isostatic Pressing. Density measurements are provided and EBSD investigations of the samples are presented. From these EBSD data, texture analysis has been performed, grain sizes determined and information on specific grains boundaries, namely Low-Angle Grain boundaries (LAGBs) and Σ3 grain boundaries data is provided. The mechanical behavior under compression tests of the processed samples is presented.

## 2 Experimental design, materials and methods

**Fig. 1:**
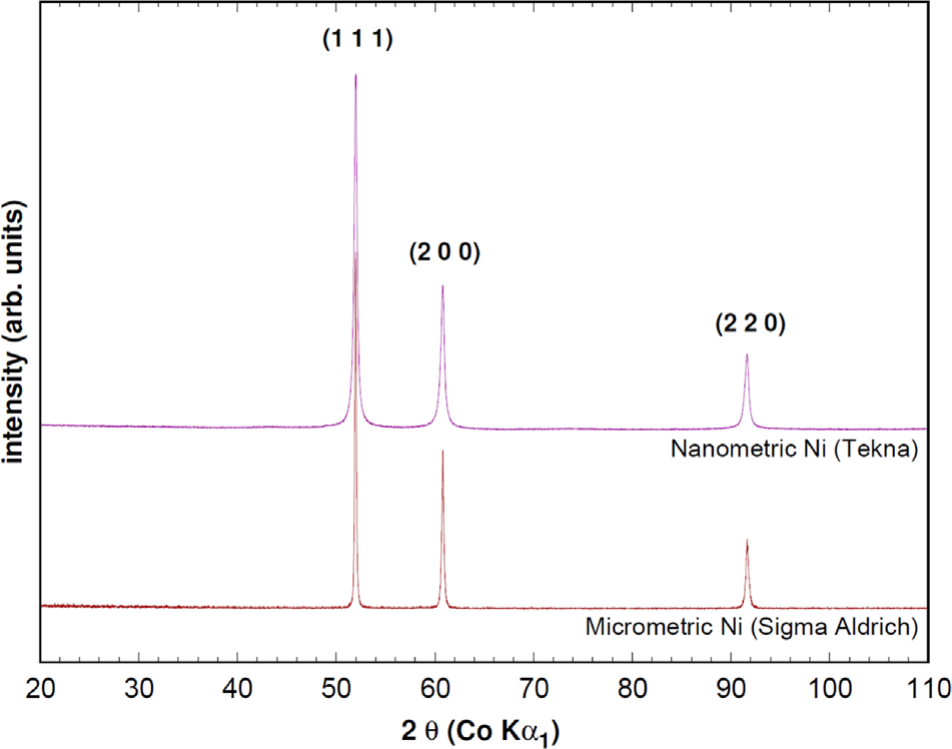
XRD patterns of the two types of powder used, showing the sole presence of Nickel.

Nanometric nickel (Ni) powder was supplied by Tekna Plasma Systems Inc. (Sherbrooke, QC, Canada). It is a monomodal powder made of spherical particles. Grain sizes were estimated by XRD using the Williamson–Hall method [1] at a value of 35 nm and by TEM, with therefore a lower statistical averaging, at 48 nm. Micrometer-sized nickel was supplied by Sigma-Aldrich (Saint Louis, MO, USA) and is made of agglomerates of about 1 µm particles, for an agglomerate size of 5–13 µm. Both initial powders were analyzed by XRD and data presented only Ni diffraction peaks, Fig. 1.

Three types of samples were prepared, from nanometric powder, from micrometer-sized powder and from a mixture made of 40% nanometric – 60% micrometric powders. For the latter, mixing was performed in a 3D Turbula mixer for 12 h before any further treatment.

Two different compaction procedures were tried, first a direct SPS sintering [2], and second using cold isostatic pressing (CIP) before performing SPS [3].

SPS was carried out by SPS using a 515S-SYNTEX machine at the regional SPS platform facility hosted by ICMPE (Thiais, France). With this setup, the maximum operating temperature is about 2273 K and the maximum pressure is about 120 MPa when a graphite die is used. The process takes place under a controlled argon atmosphere. In the present case, samples were consolidated under a 100 MPa uniaxial pressure and an electric direct current (1500 A maximum) subdivided into trains of 12 pulses separated by 2 pulses of 3.3 ms. Actually, considering instrumental limits, a uniaxial pressure of 50 MPa was first applied on the graphite die containing the powder. Temperature was then increased at a minimum rate of about 90 K min^−1^ while pressure increased up to 100 MPa. SPS cycles were performed at a temperature of 500 °C for both samples containing nanometric powders, and 850 °C for the micrometric powder in 20 mm graphite molds. The dwell time in all cases was 1 min. A previous study using a bimodal-like Ni powder (supplied by Argonide Corporation, Sanford, FL, USA) has been published previously, to which the reader might refer for experimental details [4].

This CIP operation has been performed using a pressure of 1100 MPa (11 kbar), except for the pure nanometric powder, for which a higher pressure, 1900 MPa (19 kbar) was required to ensure machinability of the green sample to perfectly fit the SPS mold [3]. Pre-compaction might be used to insert larger powder amounts inside the SPS mold. Indeed, when loose nanometric powders were used, the resulting samples had 4–5 mm in height for diameters ranging from 10 to 40 mm, whereas pre-compacted powders allowed the sintering of samples with a final height of up to 20 mm for a 20 mm diameter (10 mm height for a 10 mm diameter).

These densification procedures will hereafter be referred to as ‘direct-SPS’ and ‘CIP-SPS’, respectively.

**Fig. 2:**
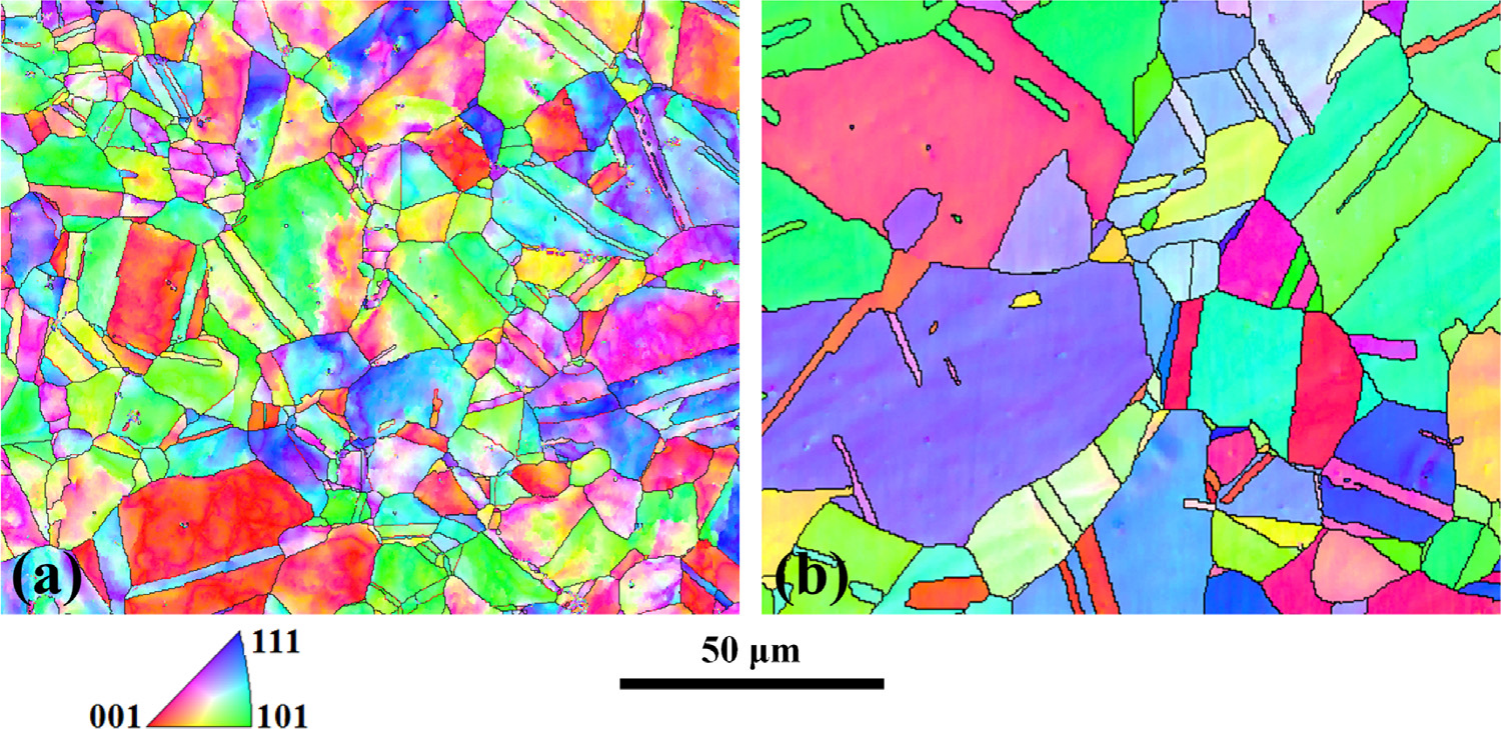
EBSD IPF images of samples sintered using SPS at 850 °C for 1 min from micrometric powders using a step size of 250 nm. (a): without pretreatment; (b): with 1.1 GPa (11 kbar) cold isostatic pre-compaction.

Fig. 2 presents the EBSD micrographs (inverse pole figures (IPF) image) obtained for the micrometric powders. Analyzing these images revealed the lack of any preferential orientation (texture). Relative densities were measured at 99% for the direct-SPS sample (a), and 98.5 for the CIP-SPS sample (b), with a relative density of the green sample of 91% after CIP at 1.1 GPa. Grains sizes were measured at 12 µm and 41 µm, respectively. Σ3 grain boundaries, including large twins, i.e. grain boundaries with a misorientation of 60±2.5° represent a fraction number of 26.6% in the direct-SPS sample, and 63.5% in the CIP-SPS sample. Low angle grain boundaries, with a misorientation lower than 15° represent then 15.6% and 3.4%, respectively. These data are summarized in Table 1 for all different samples.

**Fig. 3:**
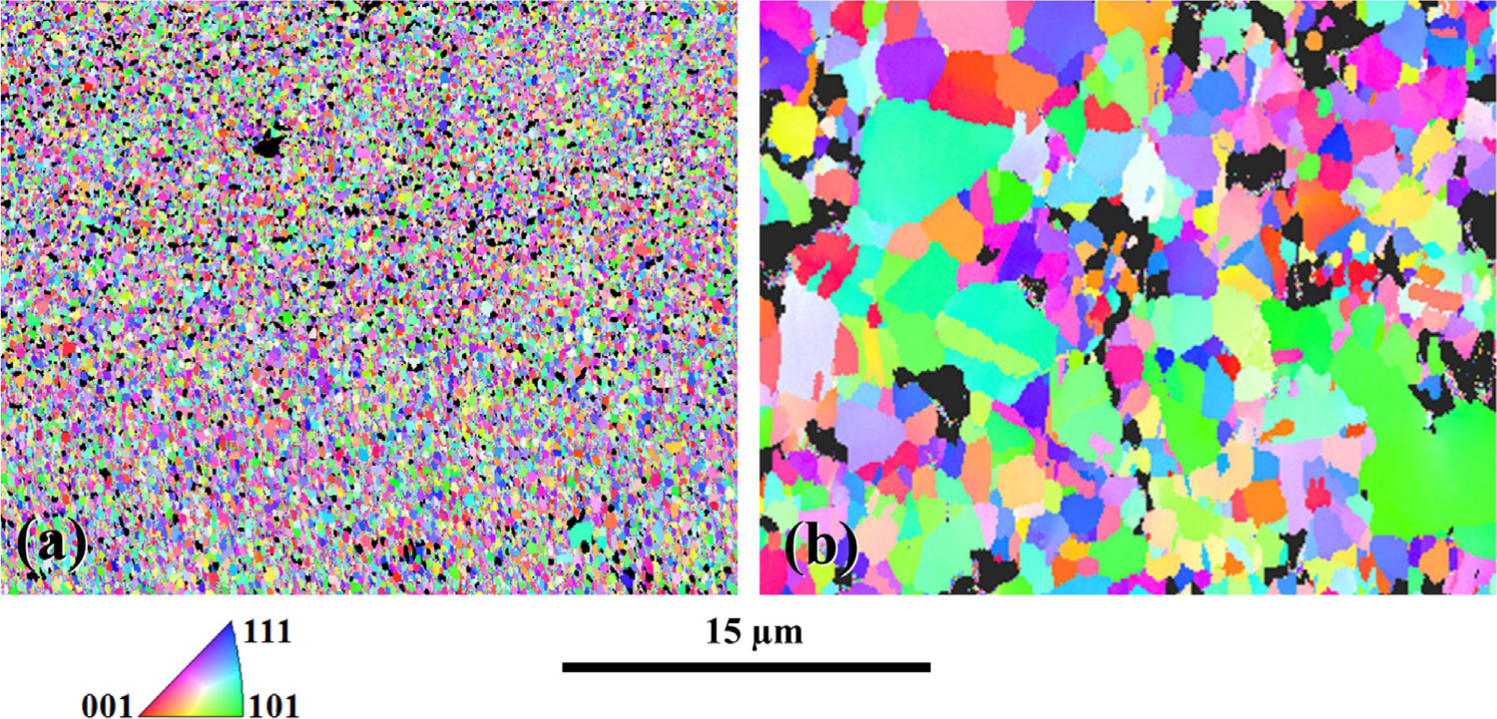
EBSD IPF images of samples sintered using SPS at 500 °C for 1 min from nanometric powders with a step size of 50 nm. (a): without pretreatment; (b): with 1.9 GPa (19 kbar) cold isostatic pre-compaction. Unindexed regions appear as black areas.

Fig. 3 presents the EBSD micrographs obtained for the nanometric powders. Analyzing these data revealed the lack of any preferential orientation. Relative densities were measured at 95.8% for the direct-SPS sample (a), and 93.9 for the CIP-SPS sample (b), with a relative density of the green sample of 87% after CIP at 1.9 GPa. Grains sizes were measured at 0.25 µm and 1.75 µm, respectively. Σ3 grain boundaries, including large twins, i.e. grain boundaries with a misorientation of 60±2.5° represent a fraction number of 20% in the direct-SPS sample, and 30% in the CIP-SPS sample. A pre-compaction appears to be a mean to increasing Σ3 grain boundaries in the processed samples. Low angle grain boundaries, with a misorientation lower than 15° represent 2% and 3%, respectively.

**Fig. 4:**
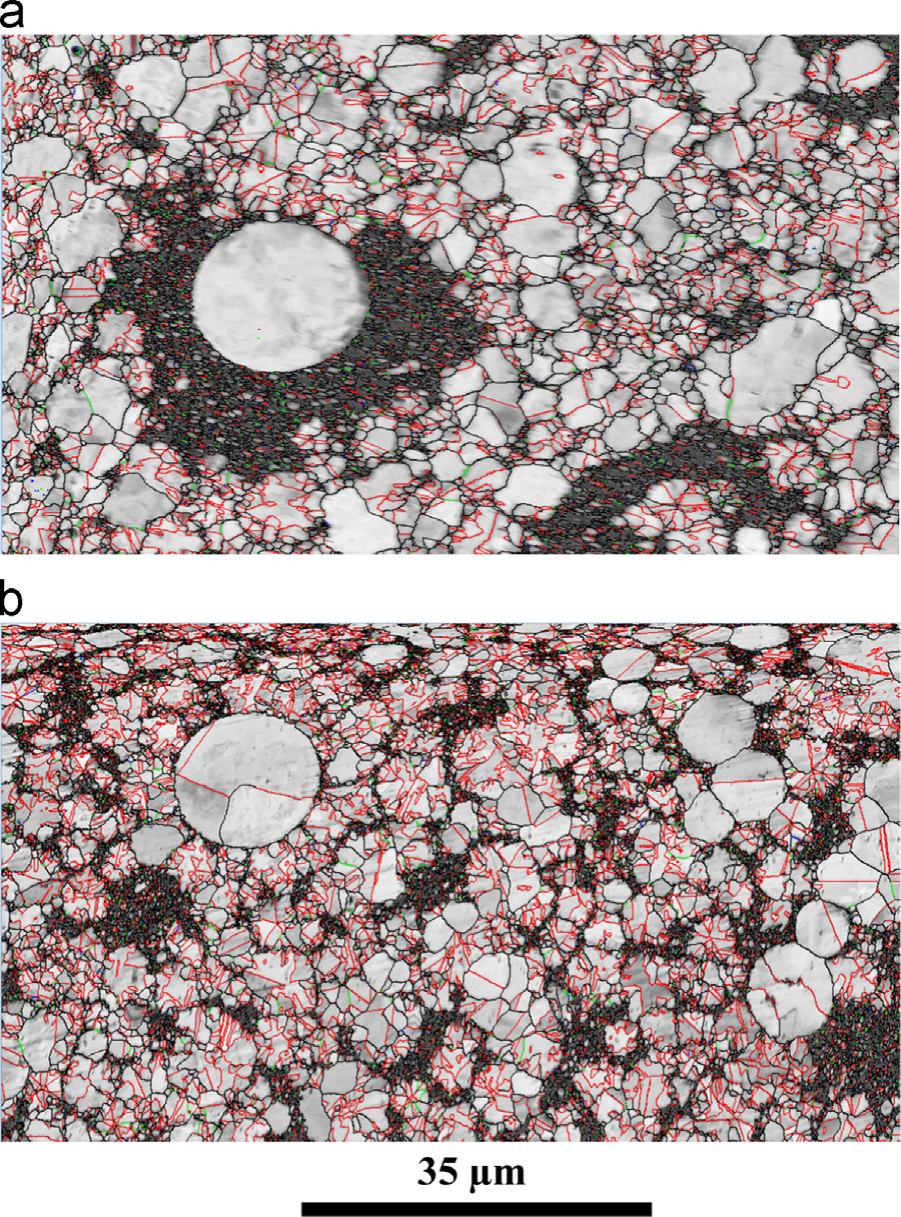
EBSD grain boundary maps images of samples sintered using SPS at 500 °C for 1 min from a mixture of nanometric (40 w%) and micrometric (60 w%) powders, with a step size of 50 nm. (a): without pretreatment; (b): with 1.1 GPa (11 kbar) cold isostatic pre-compaction. See text for more details.

Fig. 4 presents the EBSD micrographs obtained for the mixture of nanometric powders, representing 40% of the total amount, with micrometric powders, 60%. Relative densities were measured at 99.4% for the direct-SPS sample (a) and 96.3 for the CIP-SPS sample (b), with a relative density of the green sample of 87% after CIP at 1.1 GPa. After densification, no significant variation of the nanometric–micrometric volume fractions is observed, with a calculated value of 44% nanometric – 56% micrometric grains for the direct-SPS sample. Average grains sizes were measured separately for micrometric and nanometric grains. For the direct-SPS sample, the grain sizes were measured at 3.5 µm and 580 nm, respectively, and for the CIP-SPS sample, at 2.8 µm and 520 nm, respectively. Σ3 grain boundaries, including twin boundaries, are depicted in red on this figure. For the direct-SPS they represent 40% of the grain boundaries in the micrometric grains, and 17% in the nanometric ones. For the CIP-SPS sample, these proportions are 52% and 23%, respectively. Low angle grain boundaries (<15°) are represented in green. In the direct-SPS sample, they represent 1.4% in the boundaries in the case of the micrometric grains component of the microstructure, and 2.5% for the nanometric ones. For the CIP-SPS sample, these proportions are 1.4% and 3%, respectively. It can be noticed that large isolated nanometer-sized grain domains are observed for the direct-SPS processed sample, contrariwise to the case of the pre-compacted sample. In the latter case a more or less connected nanometer-size grain shell enclosing coarser grains is observed.

**Fig. 5:**
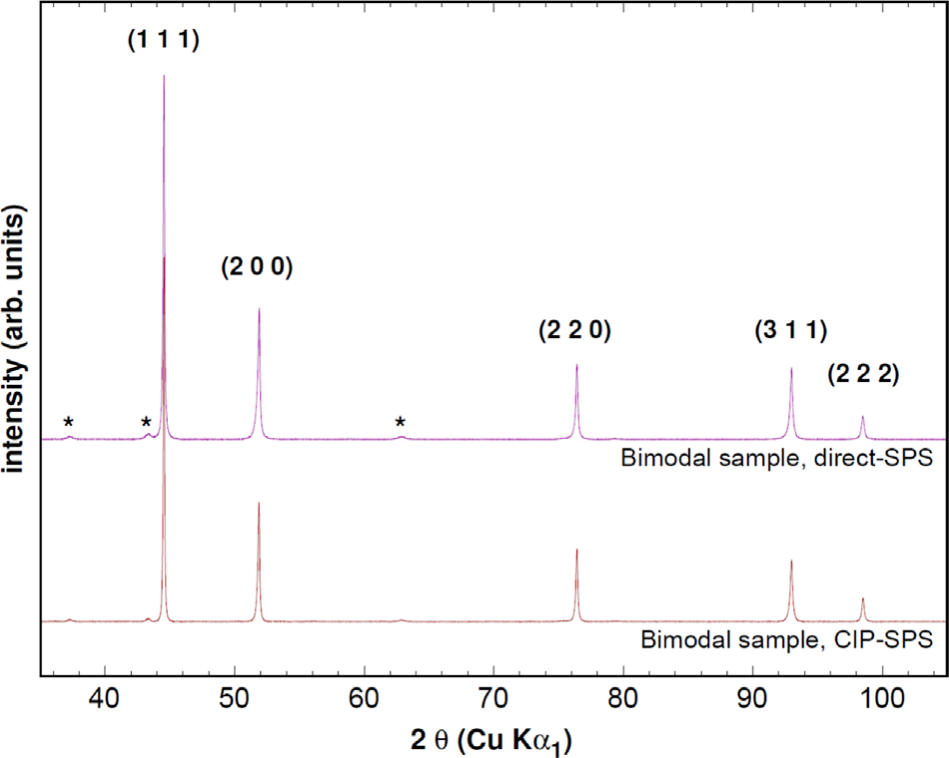
XRD patterns of the bimodal samples. (*): NiO diffraction peaks.

Fig. 5 presents the X-Ray diffraction patterns of the two bimodal samples, with and without CIP pre-compaction. In all our samples, pure Ni is by far the dominant phase, with a residual NiO phase being detected only when nanometric powders were used, as their specific surface is much higher. Rietveld analysis was performed using the MAUD program [5] and revealed a maximal NiO volume fraction of 0.0513 for the direct-SPS sample. As can be seen on this figure, peaks stay slightly larger in direct-SPS sample, due to smaller grain sizes. However, NiO contamination is 25% smaller in the CIP-SPS sample with a volume fraction of 0.0378, probably due to the fact that the powders spend less time in contact with air. These volume fractions correspond to a percentage of oxidized nickel of 0.4% and 0.3%, respectively.

Table 2 summarizes the room temperature compression data at the strain rate of 2 10^-3^ s^-^^1^ for the different samples presented here above. For the samples sintered from micrometric powders, Yield stresses of 400 MPa for the direct-SPS sample, and 200 for the CIP-SPS sample were measured. Both samples depicted a similar strain hardening behavior (not shown here), up to 50% true plastic strain, at which point the capacity of the Deben machine was reached (5000 N) and the test was interrupted.

For the samples sintered from nanometric powders, an elastic limit of 900 MPa was measured for the CIP-SPS sample. The direct-SPS sample possesses a typical brittle behavior without measurable plastic deformation before failure (therefore the data presented in Table 1 for that sample has no real meaning), while the CIP-SPS sample presents a sharp strain hardening with a measured true stress to failure of 1.57 GPa for a plastic strain of about 4%.

For the samples sintered from the 40% nanometric – 60% micrometric powders, with both compaction procedures, the elastic limit is within the 500–530 MPa range. The direct-SPS sample presents a sharper strain hardening, especially in the beginning of the plastic deformation, for a maximum stress of 730 MPa and a strain to failure of 17%. For the CIP-SPS sample, these values are 680 MPa and 34%, respectively.

## Figures and Tables

**Table 1:** Data of density measurement and EBSD observations of the different samples.

Powder type	Process type	Density (% theoretical)^a^	Grain sizes (µm)	Grain boundaries (%)	
				Σ3	LAGB
Micrometric	Direct-SPS	99	12	26.6	15.6
	CIP-SPS	98.5 (91)	41	63.5	3.4
					
Nanometric	Direct-SPS	95.8	0.25	20.0	2
	CIP-SPS	93.9 (87)	1.75	30.0	3
					
Bimodal	Direct-SPS	99.4	3.5–0.58^b^	40–17^b^	1.4–2.5^b^
	CIP-SPS	96.3 (87)	2.8–0.52^b^	52–23^b^	1.4–3.0^b^

a Density values between parentheses correspond to values measured after CIP and before SPS.

b 2 values are provided in the bimodal samples as the 2 populations are distinguishable after densification; the first value always refer to the micrometric grains, the second to the nanometric grains.

**Table 2:** Data of the room temperature compression tests of the different samples.

Powder type	Process type	Yield stress (MPa)	Strength (MPa)	Strain to failure
Micrometric	Direct-SPS	400	620^a^	> 50%^a^
	CIP-SPS	200	380^a^	
				
Nanometric	Direct-SPS	1050^b^	1050^b^	Brittle failure^b^
	CIP-SPS	900	1570	4%
				
Bimodal	Direct-SPS	530	730	17%
	CIP-SPS	500	680	34%

a The test was interrupted before failure.

b Sample failure in the elastic domain.
