# Supplementary figures and images for: Data on the influence of cold isostatic pre-compaction on mechanical properties of polycrystalline nickel sintered using Spark Plasma Sintering
Source: Data Brief. 2017 Jan 17;11:61–7. doi: 10.1016/j.dib.2017.01.009 (PMC5256669; doi:10.1016/j.dib.2017.01.009)

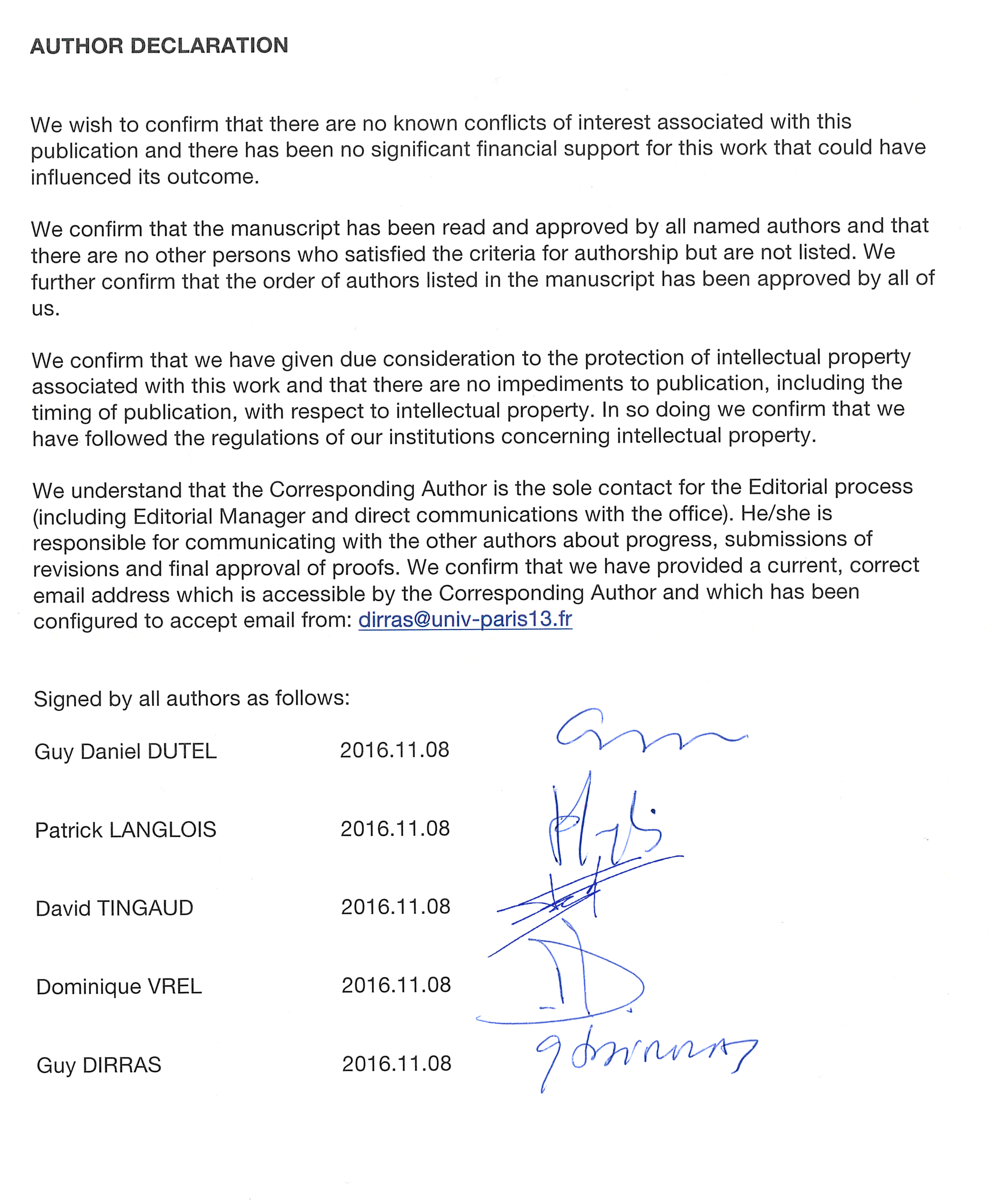

Supplement: Supplementary file 1 — Supplementary material [file mmc1.zip › mmc1.png]
